# Evaluation of the Weighted Mean X-ray Energy for an Imaging System Via Propagation-Based Phase-Contrast Imaging

**DOI:** 10.3390/jimaging6070063

**Published:** 2020-07-03

**Authors:** Maria Seifert, Mareike Weule, Silvia Cipiccia, Silja Flenner, Johannes Hagemann, Veronika Ludwig, Thilo Michel, Paul Neumayer, Max Schuster, Andreas Wolf, Gisela Anton, Stefan Funk, Bernhard Akstaller

**Affiliations:** 1 ECAP, Friedrich-Alexander-Universität Erlangen-Nürnberg, Erwin-Rommel-Str. 1, 91058 Erlangen, Germany; maria.seifert@fau.de (M.S.); mareike.weule@fau.de (M.W.); veronika.ludwig@fau.de (V.L.); thilo.michel@fau.de (T.M.); max.schuster@fau.de (M.S.); andreas.wolf@fau.de (A.W.); gisela.anton@fau.de (G.A.); s.funk@fau.de (S.F.); 2 Diamond Light Source Ltd., Diamond House, Harwell Science and Innovation Campus, Didcot, Oxfordshire OX11 0DE, UK; silvia.cipiccia@diamond.ac.uk; 3 Helmholtz-Zentrum Geesthacht, Max-Planck-Straße 1, 21502 Geesthacht, Germany; silja.flenner@hzg.de; 4 DESY, Notkestraße 85, 22607 Hamburg, Germany; johannes.hagemann@desy.de; 5 GSI Helmholtzzentrum für Schwerionenforschung GmbH, Planckstr. 1, 64291 Darmstadt, Germany; P.Neumayer@gsi.de

**Keywords:** X-ray backlighter spectrum, propagation-based phase-contrast, single-shot X-ray phase-contrast imaging, models and simulations, X-ray generators and sources

## Abstract

For imaging events of extremely short duration, like shock waves or explosions, it is necessary to be able to image the object with a single-shot exposure. A suitable setup is given by a laser-induced X-ray source such as the one that can be found at GSI (Helmholtzzentrum für Schwerionenforschung GmbH) in Darmstadt (Society for Heavy Ion Research), Germany. There, it is possible to direct a pulse from the high-energy laser Petawatt High Energy Laser for Heavy Ion eXperiments (PHELIX) on a tungsten wire to generate a picosecond polychromatic X-ray pulse, called backlighter. For grating-based single-shot phase-contrast imaging of shock waves or exploding wires, it is important to know the weighted mean energy of the X-ray spectrum for choosing a suitable setup. In propagation-based phase-contrast imaging the knowledge of the weighted mean energy is necessary to be able to reconstruct quantitative phase images of unknown objects. Hence, we developed a method to evaluate the weighted mean energy of the X-ray backlighter spectrum using propagation-based phase-contrast images. In a first step wave-field simulations are performed to verify the results. Furthermore, our evaluation is cross-checked with monochromatic synchrotron measurements with known energy at Diamond Light Source (DLS, Didcot, UK) for proof of concepts.

## 1. Introduction

Ultra-intense laser pulses focused onto solid targets produce powerful pulses of X-ray radiation, suitable for backlighting short-lived events such as strong shock propagation [1] or rapid hydrodynamic expansion [2] at Megabar pressures. Bremsstrahlung radiation generated by the energetic electrons from the relativistic laser-matter interaction reaches into the MeV spectral range [3]. However, a detailed knowledge of the emitted X-ray spectrum over a wide spectral range is elusive, as the propagation and fast relaxation of the hot electrons within the target is non-trivial. Nevertheless, the knowledge of at least the weighted mean energy of the spectrum would be of great interest for many applications. The weighted mean energy is the weighted sum of all contributing energies of a spectrum divided by the number of energy bins. The weight of each energy is given by the spectral distribution contributing to the image. The knowledge of the weighted mean energy is necessary to reconstruct high-quality phase-contrast images, which is e.g., interesting in imaging shock propagation in matter, plasmas or exploding wires. The sensitivity in the phase-contrast images of low Z materials is orders of magnitudes higher than in the attenuation images [4,5]. There are several techniques to measure the phase-contrast. One of them is Talbot–Lau interferometry. To set up a Talbot–Lau grating interferometer knowledge about the spectrum is necessary to design the gratings and the distances between the gratings correctly [6]. For phase-contrast computed tomography reconstructions, other groups [7] evaluated a so-called effective energy to be able to obtain quantitative analysis. Another technique to measure the phase-contrast is the propagation-based phase-contrast. To reconstruct propagation-based phase-contrast images of an object the weighted mean energy has to be known [8,9]. This method allows the reconstruction of the absolute phase-shift induced by an object without any optical elements, only with the help of the fringes due to the propagation of a phase-shifted wave [10].

For this purpose, we present a method to evaluate the weighted mean energy of an imaging system consisting of the X-ray backlighter Petawatt High Energy Laser for heavy Ion eXperiments (PHELIX) at Gesellschaft für Schwerionenforschung (GSI, Society for Heavy Ion Research), Darmstadt, Germany and an imaging plate Fuji BAS type SR (further specifications see Meadowcroft et al. [11]). It has to be taken into account that the weighted mean energy of the imaging system in general is not the same as the weighted mean energy of the source spectrum due to optical elements, the detector response function, etc. The presented method aims to determine the weighted mean energy of the whole imaging system including the imaging plates with a sensitivity of ±1keV. It can be used as a calibration for further measurements. It is based on the reconstruction [12] of a propagation-based phase-contrast image of a titanium wire of known thickness. The reconstructed phase is compared to the expected theoretical phase for different energies. We call the energy which leads to the best agreement of reconstructed phase and theoretical phase the dominant energy. Further, the result for the dominant energy of the imaging system is validated by wave-field simulations, simulating the propagation effects of the wire for different energies and comparing it to the measurement. The best fitting energy of the simulation should correspond to the dominant energy which can be gained by the presented method, reconstructing the phase images of the measurement. The method itself is validated by applying it at a synchrotron radiation source with a known energy of 10keV.

## 2. Materials and Methods

### 2.1. Measurement Setup at GSI

**Figure 1:**
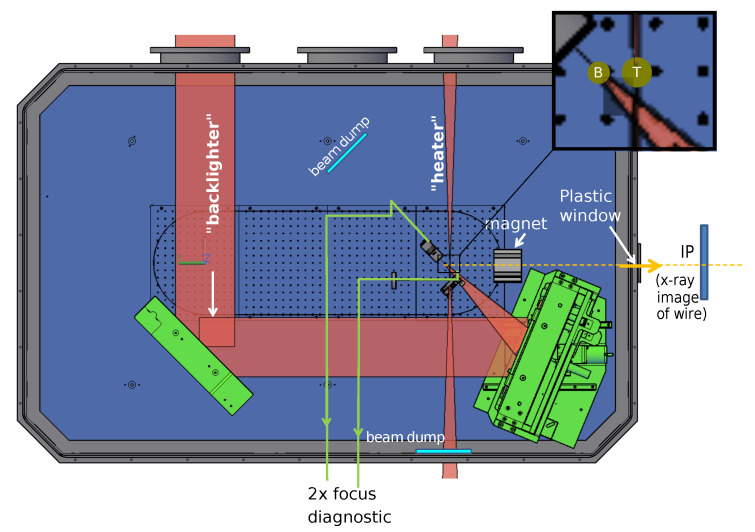
Measurement setup at Gesellschaft für Schwerionenforschung (GSI). The laser beam is split into backlighter and heater. The backlighter is shot at a tungsten wire for generating X-rays. The heater can optionally be shot at an object for generating shock waves or explosions. The inset shows a zoom-in on the tungsten backlighter wire (B) and the titanium wire (T) positions, depicted as green circles. In the presented measurement the heater is not shot at the titanium wire and the cold titanium wire is imaged. The images are obtained with imaging plates. The magnet is used to divert electrons, which occur due to the explosion of the wire and cause noise in the acquired images.

At GSI in Darmstadt, Germany, PHELIX [13] can be used to generate a short-lived X-ray source and to simultaneously generate short-living events like shock waves or explosions which are imaged by the X-ray source [14,15]. The laser system can be shot every 1.5 h and delivers two beams, namely the backlighter and the heater (see Figure 1). The backlighter targets a tungsten wire to produce X-rays. The tungsten wire is destroyed during this procedure and X-rays are emitted radially, which can be used to image an object. The heater optionally can be shot at an object for generating shock waves or explosions. This shocked or exploding object can be imaged by the radiation emitted from the tungsten wire. A tunable delay between backlighter and heater can be installed. The duration of the laser pulse is around 500fs with a pulse energy of about 50J. The whole setup is mounted in a vacuum chamber. The object is imaged by Fuji BAS imaging plates of type SR [11]. The resolution of these imaging plates was measured to be 109μm [16].

In the presented measurement the heater is not shot onto the object, instead the “cold” wire is imaged. The energy spectrum of X-rays cannot be identified by common methods because of the electron shower inside the vacuum chamber that is caused by the backlighter hitting the tungsten wire. This causes high electron noise in measurement devices such as dosimeters. Additionally, it has to be mentioned that the spectrum always differs a bit between two shots. It depends among others on the position of the tungsten target and the quality of the laser beam. Nevertheless, the estimation of the weighted mean energy is of great interest.

### 2.2. Measurement Setup at DLS

At DLS, Didcot, United Kingdom, propagation-based phase-contrast synchrotron measurements are performed. The X-ray beam was monochromated using a Si111 double crystal monochromator (bandwidth $10^{-4}$). A monochromatic energy of 10keV is set. The synchrotron beam is focused using a Fresnel zone plate with a focal length of 85mm. Three carbon fibres are placed in the cone beam at a distance of 0.105m from the focal spot. The distance of the detector to the focal spot is 14.565m. The Hamamatsu X-ray sCMOS cameraC12849 series with 6.5μm pixel size and an active zone of around $13.3\times 13.3\;{\mathrm{mm}}^{2}$ is used as a detector.

### 2.3. Propagation-Based Phase-Contrast

Propagation-based phase-contrast imaging makes use of Fresnel diffraction. There, X-rays are diffracted by an object in such a way, that intensity enhancements, also called propagation signatures, at the edges of the object in the near-field can be measured [10]. To prove whether the assumption of Fresnel diffraction is correct, the Fresnel number can be calculated. To fulfill the assumptions of Fresnel diffraction, the so called Fresnel number should be around 1. The Fresnel number (FN) can be calculated as following:

(1) $$\mathrm{FN}=\frac{d^{2}}{\lambda L},$$

with *d* the smallest feature size of the object, λ the wavelength and *L* the distance between object and detector. *d* can be assumed to be twice the projected pixel size in the object plane (e.g., [17,18]). For the following setup with the assumed weighted mean energy the Fresnel number can be calculated as 0.1. Thus, it can be assumed to be in the Fresnel regime and iterative reconstruction algorithms that are based on the Fresnel approximation can be applied [19,20].

For reconstructing the phase image of an object different phase retrieval algorithms [12,15,21,22,23,24,25,26,27] can be used. Propagation of a wave Ψ(x,y,z) in Fresnel diffraction can be modelled by the Fresnel propagator [23]

(2) $$\begin{array}{c} F(\Psi \left(x,y,z_{0}\right),z)=\\ \exp\left(ikz\right)\cdot F^{-1}\left\{\exp\left[\frac{-iz(k_{x}^{2}+k_{y}^{2})}{2k}\right]\cdot F\left\{\Psi \left(x,y,z_{0}\right)\right\}\right\}, \end{array}$$

using the plane wave approximation. Here, F denotes the Fourier transform and $F^{-1}$ its inverse. *k* is the wave number according to the weighted mean energy of the X-ray spectrum [8,9] and ($k_{x},k_{y}$) the Fourier coordinates of (x,y). The back-propagation can be computed using the inverse of *F*. If the object is placed into a cone beam, it will be magnified by the magnification *M* in the detector plane. To be able to apply the above mentioned reconstruction algorithms which hold true for plane wave approximation, the propagation distance and the detector pixel width are divided by the magnification, like in Schropp et al. [15].

In this work, the iterative phase retrieval algorithm by Clark et al. [12] is used. There, for a given energy, the wave is propagated back and forth in 1000 iterations. In each iteration the wave’s amplitude in the detector plane is replaced by the square-root of the measured intensity image. Furthermore, three constraints are applied in the image plane. First, the negativity constraint, where all phase-shifts greater than zero are set to zero. Second, the complex constraint, where the amplitude *A* is replaced by

(3) $$\begin{array}{c} A=\exp\left(\frac{\beta }{\delta }\cdot \varphi \right). \end{array}$$

Here, β and δ are the imaginary and real part of the complex refractive index, respectively, depending on energy and material properties of the object. φ is the phase of the object retrieved in the corresponding iteration. This constraint presumes that a single-material object is imaged. Finally, the wave in the image plane is restricted to the object by a mask. Thus, all wave values outside the object are set to one. The binary mask is obtained by a pre-reconstruction of the phase image by using only 100 iterations and no mask. In this pre-reconstructed image, the mask is set by hand in the image plane.

### 2.4. Computer Simulation

For the computer simulations numerical calculations are performed. An object in the beam path is represented by its transfer function. It includes the corresponding β and δ values, which are taken from Henke database [28]. The impact of the object on the wavefront is calculated using the projection approximation [29]. The propagation between the different subsystems is calculated by the band-limited angular spectrum method for numerical simulations [30]. The sampling is of the size of the detector pixel in the object plane.

### 2.5. Energy Evaluation for GSI Data

**Figure 2:**
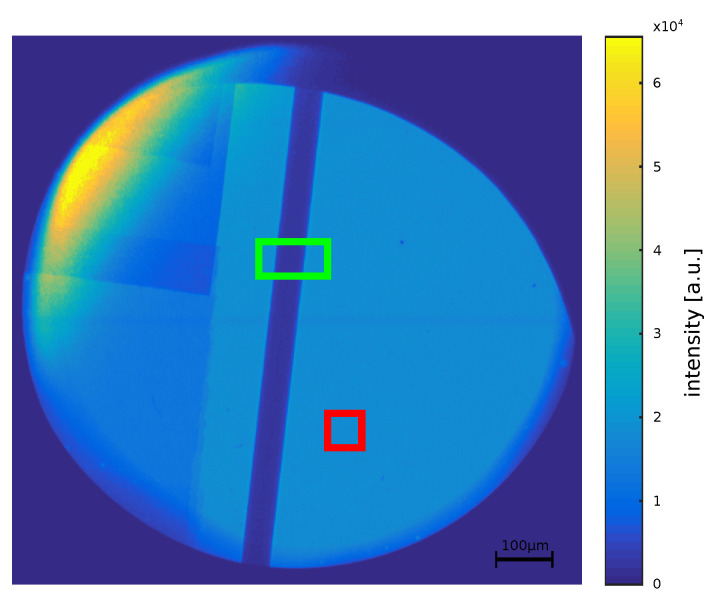
Measurement of a titanium wire with regions of interest (ROI). **Green Rectangle**: ROI that is used to reconstruct the wire. **Red Rectangle**: background.

For evaluating the dominant energy of the laser induced X-ray spectrum a propagation-based method is proposed in this study. For this purpose, the image of a titanium wire which shows edge enhancement due to propagation is used (see Figure 2). The propagation effects cannot be seen in this representation as the edge enhancement is low compared to the attenuation properties of the wire. A 5 μm tungsten wire serves as X-ray source. The 50 μm titanium wire is placed 20 mm from the source. The imaging plates are placed outside the vacuum chamber in a distance of 81.9 cm to the source. The response function of the plates has been published by Meadowcroft et al. [11]. There, it can be seen that the response does not vary substantially over an energy range of 0keV to 100keV. A maximal response of 4.5mPSL is reached at an energy of about 17keV. Nevertheless, the response between 5keV and 40keV varies between 3mPSL and 4.5mPSL. PSL is the unit of photostimulated luminiscence and describes the amount of photons which are released by the imaging plate during read-out. (Further information see Meadowcroft et al. [11].) Towards higher energies the response function decreases uniformly towards 1.5mPSL at 90keV and 1mPSL at 100keV, meaning that low energies are weighted slightly more than higher energies. Still, the response function of the imaging plates does not shift the spectrum for the evaluated energies severely.

For reconstructing the phase image of the wire in the image plane, a free-field measurement (also known as flat-field image) is needed for the reconstruction process [12] (see Section 2.3). The free-field image is determined by setting it to the mean value of a square region in the background (see Figure 2 red square). The position of this square is chosen in a rather uniform area with a certain distance to the wire and to the boundaries of the image. Multiple positions are possible which yield mean values with differences of up to 2%. However, the noise induced standard deviation is on the order of 2 to 3% of the mean value. Furthermore the alternative to choosing a background region would be to use an object-free measurement from the same detector area taken at another backlighter shot. Due to the large shot-to-shot fluctuations of the backlighter source, using a background region is preferable. In a pre-processing step, a dark-frame of the detector has to be subtracted from the object and the free-field measurement. For the imaging plates this dark-frame can assumed to be zero.

The dominant energy is evaluated in the green region of interest (ROI) shown in Figure 2. Pre-examinations have shown that the results do not change by reducing the whole image to a smaller ROI. Hence, only the ROI is evaluated in favor of computational time. The phase image is reconstructed assuming energies between 2 keV and 22 keV. Then, for each energy the mean phase-shift $\varphi_{\mathrm{reco}}$ along the wire is calculated and compared to the theoretical phase-shift $\varphi_{\mathrm{theo}}$, which is given by [23]:

(4) $$\begin{array}{c} \varphi_{\mathrm{theo}}\left(E\right)=-d\cdot k\left(E\right)\cdot \delta \left(E\right). \end{array}$$

where, *E* is the energy, *d* the diameter of the titanium wire, *k* the wave number and δ the decrement of the complex refractive index of titanium, which is obtained from the Henke database [28]. Since $\varphi_{\mathrm{reco}}$ is reconstructed using the energy-dependent refractive index, the energy dependent absorption is already regarded in this step. However the reconstruction algorithm assumes mono energetic radiation. Beam-hardening or strong absorption-edges close to the weighted mean energy of the imaging system could influence the result, but this is neglected in the evaluation.

**Figure 3:**
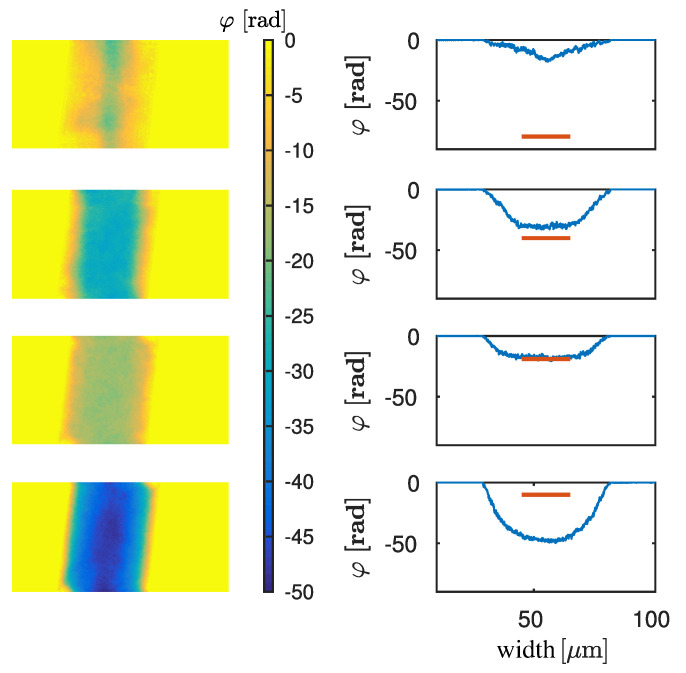
**Left**: phase reconstructions for the green marked region of interest in Figure 2 retrieved with the following energies (from top to bottom): E=[2.7;4.7;11.8;22.0]keV. **Right**: lineplots (blue) of the retrieved phase, which is shown in the **Left** images, and theoretical phase at the centre of the wire (red).

Figure 3 shows the retrieved phase images of the green ROI for four different energies, E=[2.7;4.7;11.8;22.0]keV. On the left, the reconstructed phase images of the green ROI are shown. The energy assumed for the phase retrieval increases from top to bottom. On the right a horizontal lineplot of the wire is shown in blue for the different energies. The red line shows the theoretically calculated phase-shift for the center of the wire. At the energy, which corresponds to the dominant energy, the difference between the mean reconstructed and the theoretical phase should be zero. For E=2.6keV (first row) the phase-shifts deviate significantly from the theoretical value. With increasing energy the shape of the profile becomes rounder and the values get closer to the theoretical value. For E=11.8keV (third row) the profile is round-shaped and fits the theoretical value. For E=22keV (fourth row) the phase profile is round-shaped as well, but the phase-shift at the centre of the wire differs strongly from theory.

Performing the proposed method, the mean over the phase along the titanium wire is calculated. The minimum value of the mean is compared to the theoretically assumed phase value for the given energy. The minimum of the difference of those phase-shifts (the computed one and the theoretical one) is searched. According to our definition it is the dominant energy of the imaging system. The error is calculated by dividing the standard deviation of all reconstructed lines by the square root of the number of the averaged lines.

## 3. Results

### 3.1. Evaluation of the Dominant X-ray Energy

**Figure 4:**
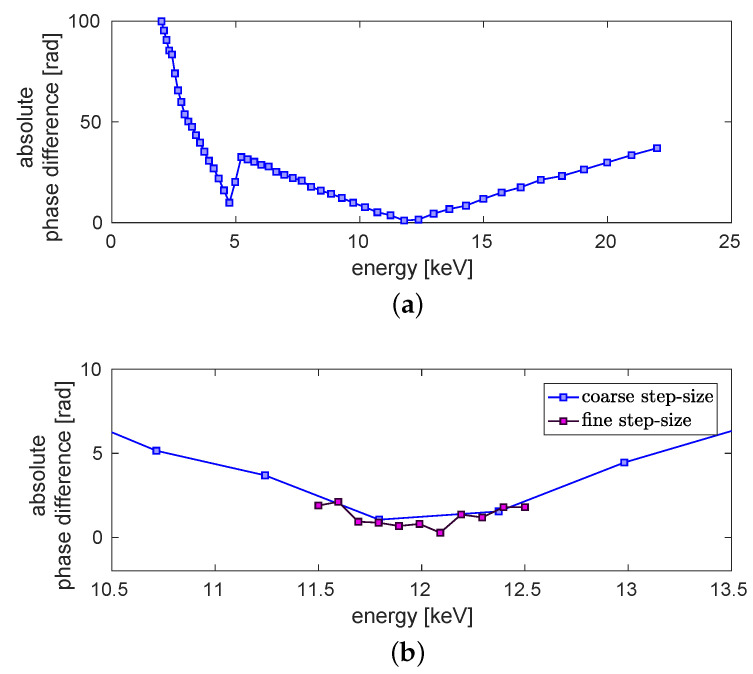
Evaluation of the dominant energy. (**a**) Absolute phase difference between theoretical phase and retrieved mean phase in dependence of the energy. The calculated energy range is between 2keV and 22keV. (**b**) Detailed view on the minimum with a finer step-size (purple). For the finer step-size the evaluated energy range is between 11.5keV and 12.5keV. In blue a section of the plot shown in (**a**) can be seen. The error is in the regime of 0.08rad.

Figure 4a shows the absolute value of the difference between the reconstructed phase of the titanium wire and the theoretical value over an energy range from 2keV to 22keV. The minimum at E=4.965keV is due to the k-edge of titanium [31]. A second minimum with an absolute phase difference close to zero can be observed for an energy around 12keV. Around this minimum the absolute phase difference is again examined with a finer step-size. The result is shown in Figure 4b (dark violet). The dark violet curve is minimal at approximately 12 keV. In blue an enlarged view of Figure 4a is shown. Thus, we conclude that the dominant energy of the imaging system is around 12 keV.

**Figure 5:**
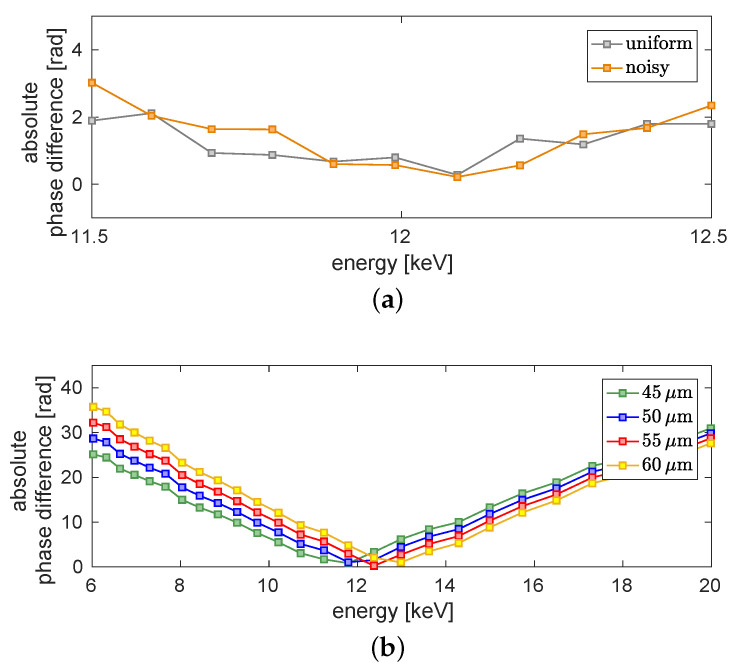
Absolute difference between the mean phase-shift of the green region of interest and the theoretical value depending on the energy. (**a**) The grey curve results from the reconstruction process with a uniform reference and the orange curve from a noisy reference with NL=31.81dB. (**b**) Results for different assumed diameters of the titanium wire. The blue curve presents the result shown in Figure 4 for the given value of the diameter of 50μm. In green, red and yellow the absolute phase difference for a theoretical phase-shift of a 45μm, a 55μm and a 60μm wire is shown, respectively. The error is in the regime of 0.08rad.

In the following, several studies are performed to show that the resulting energy is independent of initial guesses. For this purpose, the assumed uniform reference image is modified by noise. The noise level (NL) of the free-field ROI (red square in Figure 2) can be calculated as NL=31.81dB. It is added as white Gaussian noise to the uniform free-field image. Like one can see in Figure 5a the resulting phase differences (orange) of the large ROI around the minimum differs only slightly from the result acquired with a uniform reference image (grey).

The diameter of the titanium wire is specified as 50 μm. The actual diameter lies roughly in the range of 45 μm to 60 μm. The image of the wire in the detector plane has a width of 2.25 mm. Regarding only geometric magnification, this calculates to a diameter of 53.6 μm in the object plane. An uncertainty of 2.6 μm in the object plane stems from the detector pixel size of 109 μm. Further, the X-ray source size leads to about 2 μm (sigma) blurring in the object plane. In order to determine the theoretical phase-shift, the wire diameter is examined more closely in the following. For this purpose, the absolute phase difference of the green region of interest (ROI) is calculated again for three further diameters (d=[45;55;60]μm). In Figure 5b the resulting curves change slightly in direction of the energy between 11.8keV and 13.0keV by varying the diameter. The minimum of the absolute phase difference between the theoretically calculated phase and the reconstructed phase is even smaller for a diameter of 55μm than for 50μm. For a diameter of 45μm the minimal difference is 0.8rad, for 50μm it is around 1.0rad, for 55μm it is around 0.3rad and for 60μm it increases again to 1.1rad. Hence, it can be assumed that the correct diameter of the wire is around 55μm. That results in an energy of approximately 12.4keV. As the variation of the energy due to the changes of the diameter is smaller than the assumed sensitivity of ±1keV, for further evaluations still a diameter of 50μm is assumed. The determined weighted mean energy is still around 12keV. This result is identical to the variation of the assumed titanium density because a lower density is comparable to a thinner wire with the initial density. Thus, the energy evaluation is robust within the defined range of ±1keV with regard to variations of the free-field image and the wire diameter.

### 3.2. Validation of the Evaluated Dominant X-ray Energy

**Figure 6:**
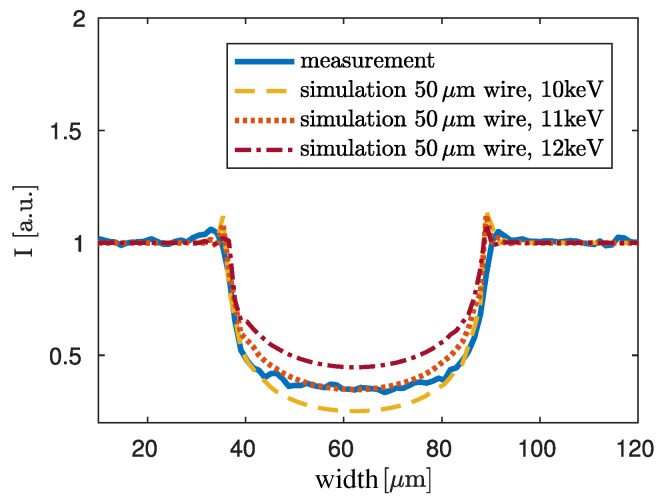
Monochromatic simulations of the propagation signature of a 50μm titanium wire for different energies. Source-blurring is neglected in the simulations. A lineplot of the measurement shown in Figure 2 is plotted in blue for comparative reasons. In yellow the simulation for 10keV, in orange the simulation for 11keV and in red the simulation for 12keV is shown. The simulation in orange for 11keV yields the best fit for the measured values at the centre of the wire.

In order to validate the result for the dominant X-ray energy, the raw image of the wire is regarded again and compared to simulation results. For this, monochromatic simulations for different energies of the propagation signature of the titanium wire have been performed. The results can be compared with the measured signatures. All simulation results are compared with a lineplot of this measurement. In a first step it is assumed that the given diameter of 50μm of the titanium wire is correct and simulations are conducted for such a wire. The simulation is performed for energies between 2keV and 22keV. It was found that the best correspondence of the simulation and the lineplot of the measurement can be found for an energy of 11keV (Figure 6, orange dotted line). The yellow dashed and the red dashed dotted line depict the results for a slightly too low energy of 10keV and a slightly too high energy of 12keV, respectively. In blue the lineplot of the measurement is shown.

For simplicity, the influence of the finite source size is not regarded in the simulations. The 5 μm source causes a blurring that can be seen best in the propagation signatures of the measurement data (blue line) in Figure 6. The edge enhancement at the boundaries of the wire are flatter but wider in comparison to the propagation signatures of the three simulated curves. Since the following evaluation is focused on the central region of the titanium wire, blurring can be neglected here.

**Figure 7:**
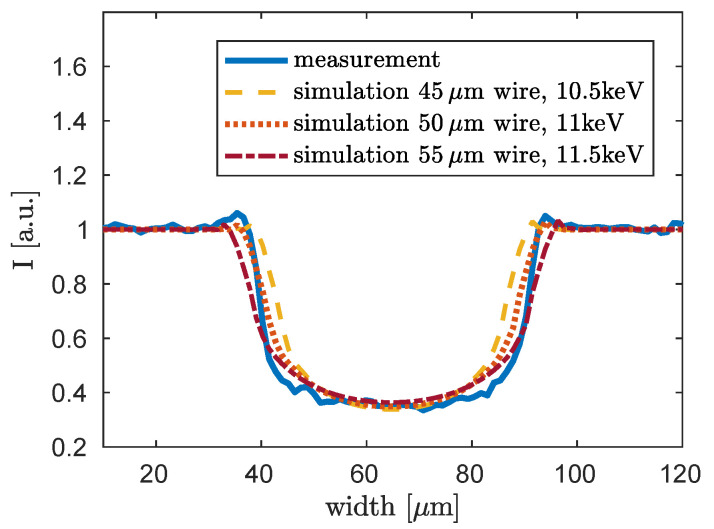
Monochromatic simulation of the propagation signature of titanium wires with varying diameter of ±10% of the supposed diameter of 50μm. The results for the best fitting energies are shown for each diameter. Source-blurring is included in the simulations. A lineplot of the measurement shown in Figure 2 is plotted in blue for comparative reasons. In yellow the simulation of a wire with 45μm diameter for 10.5keV, in orange the simulation of a wire with 50μm diameter for 11keV and in red the simulation of a wire with 55μm diameter for 11.5keV is shown.

To examine the influence of the variation of the wire’s diameter, simulations with ±10% variation of the given diameter of 50μm are performed. In Figure 7 the simulation results in comparison with a lineplot of the measurement are shown for 45μm (yellow dashed line), for 50μm (orange dotted line) and for 55μm (red dashed dotted line). The best fitting energies are 10.5keV for 45μm, 11keV for 50μm and 11.5keV for 55μm. In contrast to the evaluation shown in Figure 6, here the blurring stemming from the source size of 5 μm is implemented in the simulation, so that the width of the simulated wires can be compared to the measured line plot. The propagation signatures are not regarded here. It can be seen that a wire of 45μm is definitely too small. The correct diameter is between 50μm and 55μm, because the blue lineplot is between the orange lineplot of the 50μm wire and the red lineplot of the 55μm wire. Hence, the correct monochromatic energy for this method is between 11keV and 11.5keV. The deviation of the energy due to the diameter is within the range of the aimed sensitivity of ±1keV compared to the evaluated energy of about 12keV, which was calculated with the help of the phase image.

**Figure 8:**
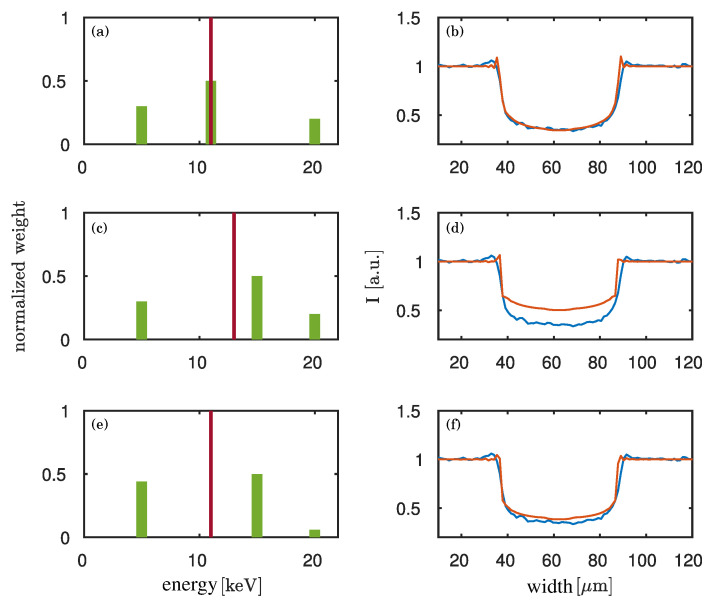
Simulation of the propagation signature of a titanium wire of 50μm diameter. On the left (**a**,**c**,**e**), three toy spectra with different weighted mean energies (red line) and with different energy distributions are assumed. Three monochromatic lines (green) at 5keV, 11keV and 20keV ((**b**)) or 5keV, 15keV and 20keV (c/d and e/f), respectively, are used to illustrate the spectra. On the right (**b**,**d**,**f**), the corresponding simulated propagation signatures of a 50μm titanium wire are shown in orange. Source-blurring is neglected in the simulations. The blue line shows a lineplot of the measurement of the wire. From top to bottom the weighted mean energy of the spectra is 11keV (a/b), 13keV (c/d) and 11keV (e/f). The maximal contributing energy bin of the spectra is 11keV (a/b), 15keV (c/d) and 15keV (e/f).

With the concept of the dominant energy the image signatures are reconstructed assuming a single energy and not a spectrum. In the following we want to investigate how far the dominant energy can be taken as an estimate for the weighted mean of the spectrum. To simulate the influence of a spectral distribution, we assume a toy spectrum with several monochromatic lines. We perform the simulation for each energy and sum up the related images. The reconstructed dominant energy of such a summed image is compared to the weighted mean of the toy spectrum. In Figure 8 assumptions for three different spectra with a weighted mean energy of 11keV (a/b and e/f) and of 13keV (c/d) are shown. Three monochromatic lines are chosen to represent the spectra with different weighted mean energies. The maximal contributing energy bin of the spectrum in (a/b) is 11keV, and the one of the spectra in (c/d) and in (e/f) is 15keV. (a), (c) and (e) in Figure 8 show the assumed spectra. (b), (d) and (f) depict the corresponding simulated propagation signatures (orange lines) and a lineplot of the measurement (blue line). It can be seen that the spectra with a weighted mean energy of 11keV (a/b and e/f of Figure 8) fit the measurement comparably well. The spectrum with a weighted mean energy of 11keV and with more influence of the low energies (e/f) shows slightly more smooth fringes at the edge of the wire due to the propagation. This corresponds better to the measurement results.

The shape of the signatures depends on the X-ray spectrum. If the high energies have a great impact on the spectrum, the fringes will be sharper as it can be seen in Figure 8 top and middle. For a wide spread energy distribution with low weights of the high energies and high weights of the low energies the fringes are blurred out (Figure 8 bottom). The angle of the deviation of the wavefront passing an object is proportional to $\frac{1}{E}$ (with *E* the energy) [23]. Thus, low energies lead to a larger deviation of the wavefront at the object’s edges than higher energies. Consequently, for low energies the edge enhancement is more widely spread.

Blurring due to the finite source size of 5 μm is neglected in the simulation data. In the presented comparison between measurement and simulation, this leads to an overestimation of the lower energies in the spectra.

With our method we determine the dominant energy of the imaging system to be 11keV. Figure 8 indicates that for an energy range of a factor 2 lower and a factor 2 higher than the dominant energy the image signatures can be reproduced by a spectrum with a weighted mean energy close to the dominant energy. This indicates that the dominant energy is a good estimate of the weighted mean energy.

### 3.3. Validation of the Evaluation Method with Monochromatic Images

**Figure 9:**
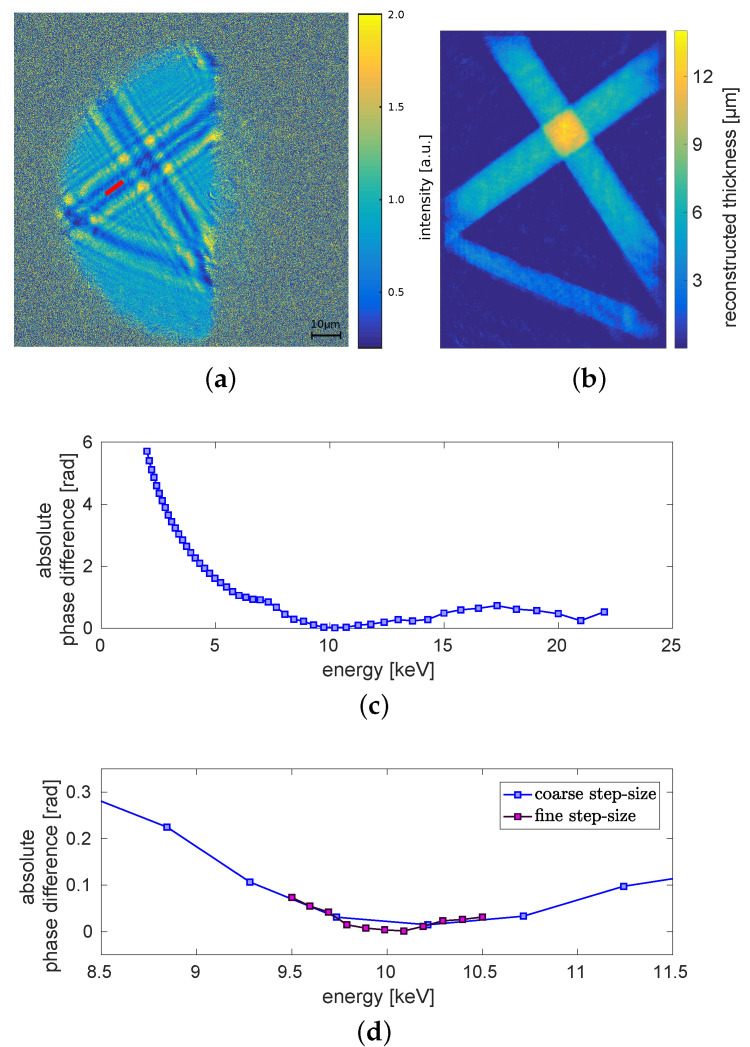
(**a**) Detector read-out of the propagation signatures of three carbon wires acquired at Diamond Light Source (DLS). (**b**) Reconstructed thickness of the carbon wires at the correct energy of 10keV. (**c**,**d**) Absolute difference between mean phase-shift of the region marked with the red line in (**a**) and theoretical value depending on the energy. (**c**) Whole energy range between 2keV and 22keV. (**d**) Energy range around the minimum between 9.5keV and 10.5keV in finer steps (purple). In blue the corresponding section of the plot in (**c**) is shown. The error is in the range of 0.01rad.

Finally, the method was tested by applying it for a measurement, which is obtained with a well-known monochromatic energy. For this purpose, a measurement acquired at Diamond Light Source (DLS), Didcot, UK, beamline I13-1 is used. The image (Figure 9a) shows the detector read-out of the propagation signature of three crossing carbon fibres. For the evaluation, the phase image is reconstructed (see Figure 9b). The mean reconstructed phase-shift is calculated along the red line at the single fibre part (see Figure 9a). The correct diameter of the carbon fibre can be calculated by reconstructing it with the correct energy of 10keV as d=6.14μm. This diameter has to be known to calculate the theoretical phase-shift. For the free-field image and the dark-frame appropriate measurements have been taken.

In Figure 9c,d the energy evaluation, which is done for the same energy range as before for the GSI measurement (compare Figure 4), is shown. The absolute phase difference curve in (c) is minimal around 10 keV as expected. The detailed evaluation with a finer energy step-size in (d) shows, that the minimum is not as sharp as the one of the measurements at GSI shown in Figure 4. The value range of the phase difference on the y-axis is much smaller. Thus, the minimum is not as pronounced as for the GSI measurements. Overall the minimum fits the known energy of 10 keV. Hence, this shows that the energy detection works well for a monochromatic beam.

## 4. Discussion and Conclusions

We were able to evaluate the dominant energy of the used imaging system at the PHELIX backlighter at GSI, Darmstadt, Germany. For this purpose, the theoretical phase-shift of a titanium wire was compared to the reconstructed phase of a propagation-based phase-contrast image for different energies. The best comparison of the phase-shifts of the theoretical value and the reconstruction was found for an energy of 12keV. This result could be confirmed by monochromatic simulations, which give a dominant energy of about 11keV for the imaging system. Hence, the combination of both examinations results in an estimation of the dominant energy of 11keV to 12keV. This is within the aimed sensitivity of ±1keV. It has to be emphasized that the result does probably not correspond to the weighted mean energy of the source spectrum, as only the spectrum convolved with the response function of the imaging system can be measured. As it can be seen in the publication of Meadowcroft et al. [11], the response function is broadly distributed over an energy range from 0keV to 100keV. The maximum of the response function is at about 17keV. It varies by not more than 50% in the energy range of 5keV to 40keV and decreases uniformly towards higher energies. Thus, we expect a small difference between the estimated weighted mean energy of the imaging system and the actual weighted mean energy of the X-ray source. Although no details about the spectral distribution are gained by the phase evaluation, the weighted mean energy of the imaging system can be estimated from the determined dominant energy. This has been qualitatively demonstrated by our toy spectra in Figure 8. Further we were able to show that the phase signatures imaged at an X-ray source with broad spectrum like PHELIX at GSI can be well reconstructed employing the concept of dominant energy.

In addition, the method was tested for monochromatic measurements which were acquired at DLS. Here, the correct energy was previously known, which could be confirmed with the presented method. This shows, that the method works properly at least for monochromatic measurements.

The knowledge of the dominant energy of the spectrum is very important for further setup designs and simulations. Additionally, to reconstruct propagation-based phase-contrast images of unknown materials, it is important to know the dominant energy of the spectrum. With the presented method a type of calibration can be performed for unknown X-ray sources in preparation of further measurements.
